# Sexual and Dating Violence Prevention Programs for Male Youth: A Systematic Review of Program Characteristics, Intended Psychosexual Outcomes, and Effectiveness

**DOI:** 10.1007/s10508-023-02596-5

**Published:** 2023-05-24

**Authors:** Mirthe Verbeek, Joyce Weeland, Maartje Luijk, Daphne van de Bongardt

**Affiliations:** Youth and Family, Department of Child Development and Education, Erasmus University Rotterdam, P.O. Box 1738, 3000 DR Rotterdam, The Netherlands

**Keywords:** Sexual violence, Sexual education, Adolescent boys, Sexual health, Structured literature review

## Abstract

Sexual and dating violence (SDV) by male youth (≤ 25 years)—including sexual harassment, emotional partner violence, and rape—is a worldwide problem. The goal of this preregistered (PROSPERO, ID: CRD42022281220) systematic review was to map existing SDV prevention programs aimed at male youth, including their characteristics (e.g., content, intensity), intended psychosexual outcomes, and empirically demonstrated effectiveness, guided by the principles of the theory of planned behavior (TPB). We conducted searches in six online databases for published, peer-reviewed quantitative effectiveness studies on multi-session, group focused, and interaction based SDV prevention programs for male youth ending March 2022. After screening of 21,156 hits using PRISMA guidelines, 15 studies on 13 different programs, from four continents were included. Narrative analysis showed, first, broad ranges in program intensity (2–48 h total), and few program curricula included explicit discussion of relevant aspects of the TPB. Second, programs’ main intended psychosexual outcomes were to change SDV experiences, or related attitudes, or norms. Third, significant effects were found mostly on longer term behaviors and short-term attitudes. Other theoretical proxies of SDV experiences, such as social norms and perceived behavioral control, were sparsely investigated; thus, program effectiveness on these outcomes remains largely unknown. Assessed with the Cochrane Risk of Bias Tool, moderate to serious risk of bias arose in all studies. We present concrete suggestions for program content, such as explicit attention to victimization and masculinity and discuss best practices for evaluation research, including assessments of program integrity, and examining relevant theoretical proxies of SDV.

## Introduction

Sexual and dating violence are persistent problems all over the world (Borumandnia et al., 2020; Rubio-Garay et al., 2017; World Health Organization (WHO), 2021). Sexual violence includes “any unwanted sexual activity where consent is not received or freely given, which can occur within romantic relationships but also between acquaintances or strangers” (Graham et al., 2021) and dating violence consists of psychological, physical and/or sexual violence between adolescent dating partners (Center for Disease Control & Prevention, 2020; Wekerle & Wolfe, 1999). Male youth are at an increased risk of developing these types of behavior (Basile et al., 2009; De Bruijn et al., 2006; Foshee et al., 2001), suggesting that the periods of adolescence and young adulthood (i.e., up to 25 years old) are promising times of opportunity for effective prevention. Hence, the aim of the current study is to synthesize the scientific knowledge regarding characteristics, intended psychosexual outcomes and effectiveness of sexual and dating violence prevention programs for male youth.

### Sexual and Dating Violence in Youth

As shown by a systematic review of worldwide prevalence studies among adolescents and young adults, percentages of victimization of physical dating violence among youth can go up to 57.3%, and sexual violence up to 64.6% (Rubio-Garay et al., 2017). First experiences with sexual and dating violence (hereafter termed SDV) often occur when victims are under 25 years old. For instance, a large representative population study in the USA found that most intimate partner violence occurred between the ages of 18 and 24 years (Breiding et al., 2014). Moreover, a study on a large sample of Dutch tertiary education students found that 23% of students first experienced sexual violence before commencing their studies (Driessen & Polet, 2021).

Experiences with SDV can generally have severe and long-lasting effects on victims’ mental, physical and (sexual) health and wellbeing, such as depression, anxiety, sexually transmitted infections, alcohol abuse and problems with fertility (Choudhary et al., 2012; WHO, 2013). Moreover, victimization of sexual or dating violence in youth specifically—up to 20 years old—has been linked to reduced academic achievement, lower self-esteem, and longitudinal transmission of experiences with sexual and intimate partner violence into later romantic relationships as shown by longitudinal as well as retrospective research (Driessen & Polet, 2021; Offenhauer & Buchalter, 2011, respectively). Therefore, it is important that researchers, practitioners, and policymakers focus on developing and implementing early, and effective strategies for the prevention of SDV.

### A Focus on Young Men

Traditionally, psychosexual health education has typically been investing a lot of effort in making girls and young women more “resilient” against SDV (Mahoney et al., 2020). Yet, logically, the most promising way of preventing SDV experiences/victimization is to prevent its perpetration (Harvey et al., 2007). Although both men and women can be perpetrators and victims of SDV, various large prevalence studies among youth as well as adults indicate that girls and women are more often victimized, whereas boys and men are more often perpetrators (De Graaf et al., 2017; Driessen & Polet, 2021; Rubio-Garay et al., 2017). Moreover, SDV perpetrated by boys and men has more negative effects on its victims than SDV perpetrated by girls and women in terms of subsequent severity of potential injuries, emotional trauma, and fear (Archer, 2000; Garcia-Moreno et al., 2013; Wekerle & Wolfe, 1999). Hence, for SDV perpetration prevention, it is crucial to target men.

Three systematic reviews investigating sexual violence prevention for men, including both youth and adults, found that the only programs for which there was substantial evidence of effectiveness to reduce SDV perpetration, were those focused on adolescents (i.e., maximum 18 years old) compared to those for undergraduate/college students or adults (DeGue et al., 2014; Graham et al., 2021; Ricardo et al., 2011). In line with this, a meta-analysis on adult men found no evidence for reduced perpetration (Wright et al., 2020). Together, these findings indicate the importance of focusing SDV prevention programs on male youth (Schneider & Hirsch, 2020). Consistent with renowned developmental researchers (Sawyer et al., 2018), as well as the United Nations (2018) stating that in many parts of the world, developmental adulthood is not reached until the age of 25, we use the term ‘youth’ to describe the broad range of young people aged 10 ≤ 25 years of age. This age cut-off is also used by large population studies on youth sexual health (Buysse et al., 2013; De Graaf et al., 2016, 2017).

### Understanding Sexual and Dating Violence Through the Theory of Planned Behavior

**Fig. 1:**
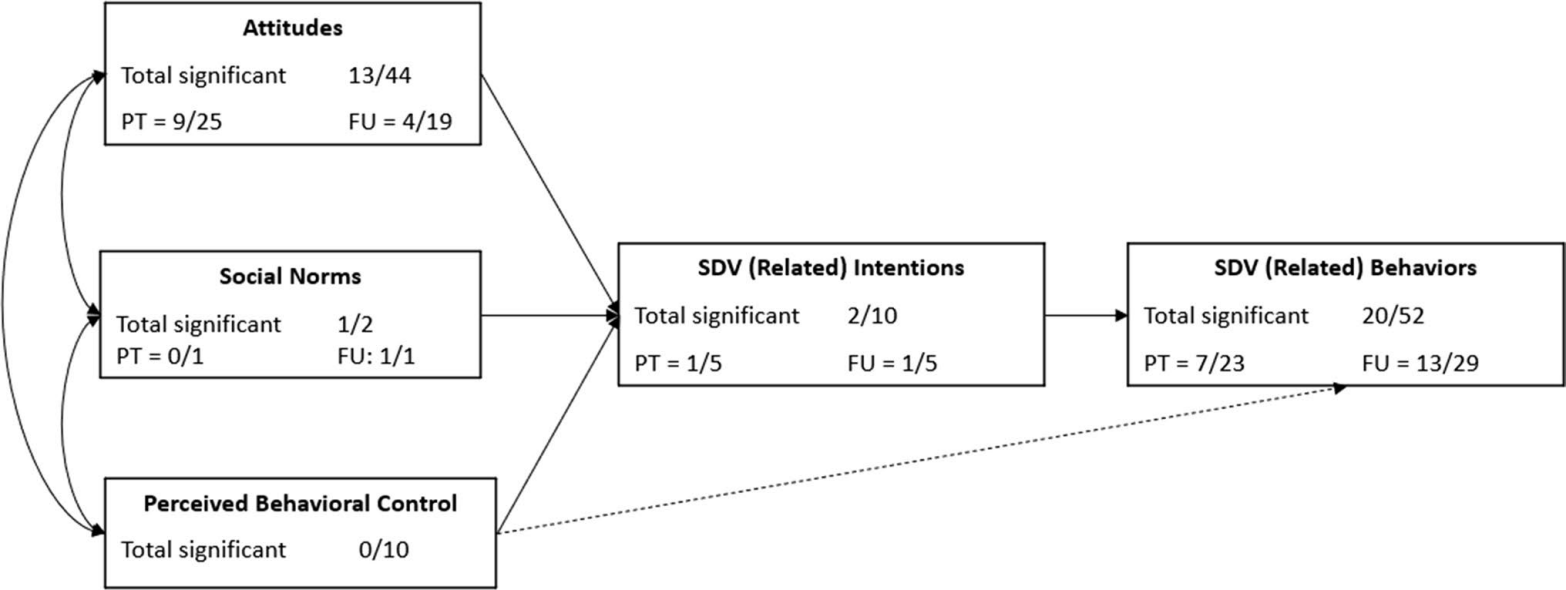
Number of significant effects per outcome type of the TPB. *Note.* PT = post-test, FU = follow-up

One theory that provides a framework for the relevant antecedents of SDV that prevention may focus on, is the theory of planned behavior (TPB) by Ajzen (1991). The TPB states that more positive attitudes towards a certain behavior, perception of approving social norms regarding that behavior, and better (perceived) behavioral control to perform the behavior are all associated with higher intentions and, subsequently, higher chances of performing the behavior (see Fig. 1). Combining individual, as well as sociocultural factors to explain behavior, this theory has already commonly been used to explain experiences with SDV (Miller et al., 2010), also among youth. For instance, a study among university students in Taiwan found that multiple TPB concepts, including positive attitudes towards dating violence, positive perceived injunctive norms regarding SDV, and higher perceived behavioral control to perform violence against dating partners were all related to dating violence perpetration (Lin et al., 2021a). This theory can also be used to explain susceptibility for experiencing SDV as a victim, as a study on Chinese adolescents found that for boys, higher perceived behavioral control and more positive social norms towards rejecting peers’ sexual assault were related to more behavioral intentions to reject peers’ sexual assault (Li et al., 2010).

Several other studies have also shown that the specific concepts of the TPB are overrepresented in young men and are indeed linked to SDV perpetration by young men. First, male youth have been found to develop attitudes conductive to SDV. For instance, a recent study on Dutch adolescents showed that especially adolescent boys (but not girls) pick up gender inequitable attitudes when presented by media and peers (Endendijk et al, 2022). Moreover, adolescent boys in the Netherlands as well as China who endorsed gender inequitable attitudes and attitudes justifying dating violence, were more likely to engage in sexual and physical dating violence perpetration (De Bruijn, et al., 2006; Shen et al, 2012). Second, both injunctive norms accepting of sexual (risk) behavior and descriptive peer norms indicating more sexual (risk) behavior by peers, have been found to be related to youth’s higher sexual (risk) behavior and sexual activity (meta-analysis: Lin et al., 2021b; Van de Bongardt et al., 2015). More specifically, regarding descriptive norms, boys in the USA who perpetrated dating violence were more likely to believe that their friends also perpetrated dating violence themselves (Reed et al., 2011).

### Theory of Planned Behavior in Sexual and Dating Violence Prevention Programs

While it seems to be a sensible strategy to aim SDV prevention efforts at male youth, and to also target relevant TPB-factors associated with SDV experiences, an integrated overview of the availability and success of such programs is currently missing. Although some SDV prevention strategies have actually been specifically designed based on the TPB, by targeting attitudes, social norms and perceived behavioral control (Cotto-Negrón, 2019; Montanaro & Bryan, 2014), this is more often not the case. A systematic review (DeGue et al., 2014) on what works in sexual violence prevention strategies, which included mostly programs for college students and adult men, concluded that most programs focused mainly on knowledge about sexual violence or the laws prohibiting it, while these are neither theory-based, nor indicated by empirical evidence as significantly predicting SDV behaviors. In turn, factors such as traditional gender role attitudes and attitudes that men need to be dominant and aggressive (i.e., relevant attitude-factors from the TPB) have shown consistent links to sexual violence (Tharp et al., 2013). Yet, these were embedded in only two of the 128 included programs in the review by DeGue et al. (2014), of which one was intended for male youth. As it is not yet known how and to what extent relevant theory-based factors from the TPB are embedded in the intended psychosexual outcomes and curricula of SDV prevention programs for male youth, the first aim of the current review is to map the characteristics, content and intended psychosexual outcomes of these programs.

### Research on Effectiveness of Sexual and Dating Violence Prevention Programs

Previous systematic reviews and meta-analyses on SDV prevention have been conducted among two themes. One theme is focused on dating violence prevention for youth in general (i.e., for both boys and girls). The other theme is focused on SDV programs for men with no age specification (i.e., both youth and adults).

Regarding the first theme, dating violence among adolescents, previous systematic reviews (De La Rue et al., 2014; De Koker et al., 2014) and meta-analyses (Edwards & Hinsz, 2014; De La Rue et al., 2017; Lee & Wong, 2020; Russel et al., 2021; Ting, 2009) found that these often school-wide, universally implemented programs appear effective in improving attitudes regarding dating violence (De La Rue et al., 2014, 2017; Lee & Wong, 2020; Ting, 2009) and skills (De La Rue et al., 2014). However, one meta-analysis found no effects on skills and attitudes (Fellmeth et al., 2015). Moreover, some systematic reviews investigating randomized controlled trials (RCTs) on adolescent dating violence prevention programs, indicated that these programs may reduce perpetration of dating violence, as well as dating violence victimization (Edwards & Hinsz, 2014; Russel et al., 2021; Lee & Wong, 2020; De Koker et al., 2014). However, a meta-analysis found inconclusive results about program effects on perpetration of SDV (De La Rue et al., 2017).

Despite some inconsistencies in their findings, these studies have provided valuable insights on the promising efforts to prevent SDV among youth. Yet, an important limitation of these previous reviews and meta-analyses is that they all focused on violence between dating partners. Considering the broad spectrum of SDV (including SDV among non-dating partners, such as making sexual comments) is important, because especially in youth, this behavior may develop into more serious forms, including intimate partner violence, over time (Wekerle & Wolfe, 1999; Espelage et al., 2014; Cutbush et al., 2016). If we do not consider the possible cross-over of these experiences, we might miss possibly vital opportunities for SDV prevention. Moreover, a sole focus on program effects on behavioral outcomes may not be the most fitting in youth, as some types of SDV (e.g., physical sexual coercion or rape) can only occur after sexual debut. Many may not yet be sexually experienced (De Graaf et al., 2017) at the time they receive the program making it difficult to prove behavioral change as the result. Therefore, focusing not only on behavioral outcomes, but also on the relevant antecedents of this behavior, will provide more rich information for future research on and development of prevention efforts for male youth.

Regarding the second theme, two systematic reviews (DeGue et al., 2014; Graham et al., 2021) examined SDV programs for men in general (combining both youth and adult samples), and two meta-analyses (Anderson & Whiston, 2005; Wright et al., 2020) investigated SDV prevention programs for adult men. One additional systematic review did focus on young men (12–19 years old), the main difference with the current review being that this review also included mixed-gender programs (Ricardo et al., 2011). In contrast to systematic reviews and meta-analyses of the first theme all focusing on dating violence among youth, three of the systematic reviews and meta-analyses of the second theme focused only on sexual violence in general but not dating violence (Anderson & Whiston, 2005; DeGue et al., 2014; Wright et al., 2020). Together, these studies suggest positive effects on bystander behaviors (DeGue et al., 2014; Wright et al., 2020), SDV related intentions (Anderson & Whiston 2005; DeGue et al., 2014; Wright et al., 2020), attitudes (Anderson & Whiston 2005; Ricardo et al., 2011), and skills including communication and bystander intervention skills (DeGue et al., 2014). However, null effects were also common, and some empirical studies even found negative effects in terms of increased rape proclivity or increases in SDV perpetration (DeGue et al., 2014).

Concluding, in addition to the aforementioned gap in knowledge about program content regarding whether the factors from the TPB are embedded in SDV prevention programs, it is not yet known whether these programs in turn, are effective in changing these factors. In addition, most of these prior reviews and meta-analyses did not have any requirements for the type of program (e.g., a one-time video presentation, or a 10-week group counselling program) or type of delivery style (group discussions versus a theatre show), resulting in inconclusive results and questions about possible differentiation between programs with different intensity and work forms.

### The Current Study

The overarching aim of this study is to synthesize the existing scientific knowledge about the characteristics, intended psychosexual outcomes and effectiveness of SDV prevention programs specifically developed for male youth (i.e., up to 25 years old), aimed at the prevention of the broad spectrum of SDV. In doing so, we will describe the effectiveness of these programs regarding SDV experiences, and the theoretical proxies of SDV experiences according to the TPB (i.e., attitudes, social norms, (perceived) behavioral control and intentions). The second aim is to gain insight into the characteristics and quality of the effectiveness studies evaluating these programs, including their design, and investigated outcomes. We focused specifically on group focused, multi-session, and interaction-based programs, as it has been suggested that these ingredients are most often related to effectiveness (Anderson & Whiston, 2005; De Koker et al., 2014; Ricardo et al., 2011), and this focus will inform us on more specific knowledge about the effectiveness of the currently known most promising type of program. Lastly, current practice as well as research is focusing mostly on boys and (young) men and SDV perpetration (DeKeseredey et al., 2017). However, it is becoming increasingly clear that SDV victimization can also be experienced by boys and (young) men, and even be a precursor of perpetration (De Bruijn et al., 2006; Jennings et al., 2012; Rubio-Garay et al., 2017). As such, we will explore whether victimization receives any attention in the programs as well as the studies evaluating these programs when aimed at male youth.

## Method

### Study Selection Criteria

**Fig. 2:**
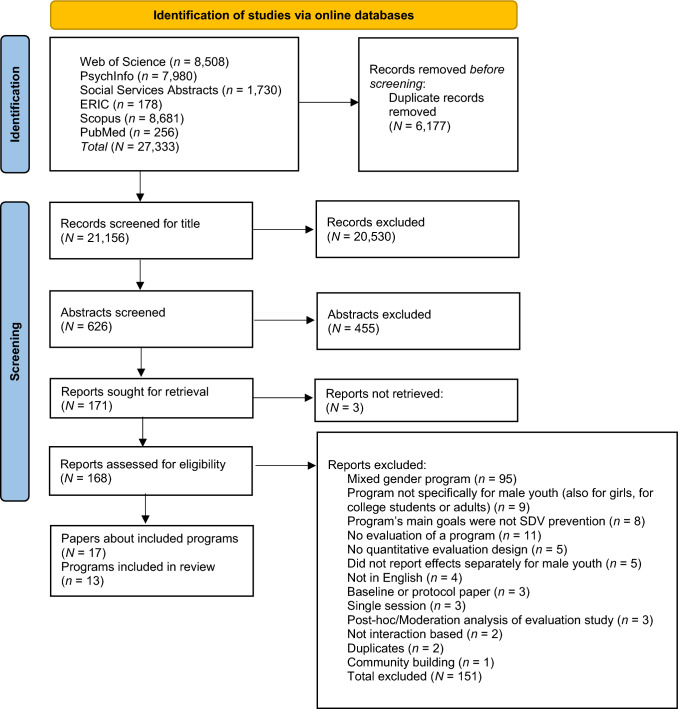
PRISMA flow diagram of study selection

We had selection criteria for both the empirical evaluation studies, as well as the SDV prevention programs that were evaluated in these studies. For the studies, we used four criteria:

1. They evaluated programs aimed at either preventing SDV perpetration and/or victimization experiences of male youth before they occur (i.e., primary, or universal programs) or intervening in youth with specific risks to perpetrate SDV (i.e., secondary, or selective programs). We excluded: Broad sexuality education or masculinity programs that were not specifically aimed at preventing SDV and treatment programs for previous offenders/SDV perpetrators.
2. They had a quantitative design, such as a (cluster) randomized controlled trial (RCT), quasi-experimental design, or pre-posttest evaluation without a control group.
3. They assessed experiences with SDV perpetration and/or victimization, and/or one or more TPB proxies of these experiences, broadly including all SDV-related attitudes, norms, perceived behavioral control (e.g., skills or self-efficacy) or behavioral intentions. This inclusion criterium did not result in the exclusion of any records (see Fig. 2).
4. They published in peer-reviewed journals in English language (no restrictions on year of data collection, publication date or study location).

For the evaluated programs in the studies, we used three criteria.

1. We included programs that were aimed at male youth (i.e., mean age of the participants could be max. 25 years old). Participants could be school-going, living in youth-care facilities, visiting community centers, or working. Participants could also be youth with mild psychiatric- or behavioral problems.

We excluded programs that had one or more mixed-gender sessions, and programs specifically focused on college- or undergraduate students as the effectiveness of these programs has already been evaluated extensively (Anderson & Whiston, 2005; DeGue et al., 2014; Graham et al., 2021; Ricardo et al., 2011; Wright et al., 2020), they are adults, and college students encounter specific risk factors for perpetrating sexual violence, such as fraternity membership (Murnen & Kohlman, 2007) that do not apply to the general population.

(2) Based on previous reviews indicating the following to be the most promising type of program, we included: protocolled programs in which the delivery mode was in person, group focused, and interaction based. This means that the program should have at least one program facilitator and at least two young male participants, who interact with each other and the person who delivered the program.

We therefore excluded programs that did not meet these criteria, such as (theater) presentations, film depictions, parent-delivered programs, broad community interventions without a pre-specified program, and fully digital or online interventions.

(3) Also based on previous reviews indicating the following to be the most promising type of program we included programs consisting of at least two program sessions.

We therefore excluded programs that did not meet this criterium, such as single-session prevention strategies, and programs without a specified number of meetings.

### Search Strategy

We conducted a systematic literature search using PRISMA guidelines (Page et al., 2021), see Appendix 1. To find studies that matched the inclusion criteria, six online databases were searched that were expected to result in the most relevant hits about the current topic: Web of Science, PsychInfo, Social Services Abstracts, ERIC, Scopus, and PubMed. For the database-specific search terms and filters that were used, see Appendix 2. The protocol for this systematic review was registered on PROSPERO (ID: CRD42022281220). The literature search started in March 2021 and was updated in March 2022.

### Selection Process

The online database searches resulted in 21,156 hits after duplicates were removed. The selection process is depicted in Fig. 2. No automation tools were used in the selection process. The first author screened all titles for remote relevance to the current review. When a title was deemed possibly relevant, the abstract was screened as well. For these first two steps, broad criteria were applied (i.e., title/abstract mentions description/evaluation of a program on a sexual or dating/relationship violence related topic). Next, the full texts of the relevant articles (*k* = 169) were retrieved and screened for eligibility based on the inclusion and exclusion criteria. Any doubts about the eligibility of full-text articles were discussed among the authors to achieve consensus (*k* = 23). The full-text articles that met all criteria, were included.

### Data Collection

Relevant data of all selected articles was coded and entered in a structured Excel sheet that was based on the coding scheme used by DeGue et al. (2014) by the first author and a trained research assistant. Both researchers coded all variables of all included papers, and differences were sought and resolved to achieve a 100% certainty of all coded variables. The relevant data from the included studies that was entered in the Excel sheet, were used to synthesize the results in the text, figures, and tables. If information on statistical outcomes was not reported in a paper, we contacted the authors. In total, we contacted six authors of *k* = 11 papers to request additional data on program implementation and effectiveness statistics. Two authors responded and provided the requested information. Three authors responded but could not provide the requested information. From one author, no response was received.

### Coded Study and Program Variables

Data were coded on program characteristics, characteristics of the included studies, and program implementation, the programs’ intended psychosexual outcomes and effectiveness according to the TPB. The coding scheme is available upon request to the authors.

#### Characteristics of the Sexual and Dating Violence Programs

Regarding program characteristics, we coded the program name, program delivery (e.g., active participants using role plays versus mostly lectures), gender of program facilitators, type of facilitators (e.g., peers versus adults, untrained versus trained), the target groups (e.g., universal or specific populations), session length, and number of core sessions, intended psychosexual outcomes, and program content. We also coded program integrity characteristics in terms of dosage (i.e., how many program sessions participants joined), and adherence (i.e., how much of the program was executed by the facilitators as intended from the protocol). It should be noted that dosage and adherence are partly program characteristics and partly study characteristics. For instance, adherence possibly indicates how easy it is to follow the protocol—a program characteristic -, but how much of the program facilitators executed can also depend on other factors such as time of year—a study characteristic.

#### Characteristics of the Evaluation Studies

Regarding characteristics of the included effectiveness studies, we coded sample size, retention rate, sample characteristics (age, ethnicity/racial background), study location (e.g., country, urban versus rural), site of the program implementation (e.g., school, community), study design, number of measurements and time between measurements, and type of comparison group (e.g., wait-list control group or none).

#### Psychosexual Outcomes of the Sexual and Dating Violence Programs

We double-coded all relevant statistical results of all relevant SDV related psychosexual outcomes related according to the TPB. Statistics coded were means and standard deviations, percentages, medians and interquartile distances for behaviors as they were reported in the studies. Moreover, we coded significance of the analyzed differences between experimental- and control groups at post-test and follow-up, or between the pre- and post-test measurement. This was done separately for each outcome from each study.

If data for effect size calculation were not readily available in the manuscript, we contacted the authors to retrieve missing data. Some randomized controlled trials (RCTs) or quasi-experimental studies also reported within-person analyses. For comparability across similar studies and because most RCTs and quasi-experimental studies only reported intervention effects (i.e., comparisons between intervention and control group), we did not take into account these analyses.

### Bias Assessment

To assess the quality of the included studies, we used Elridge et al. (2021) Revised Cochrane Risk of Bias tool for assessing the risk of bias in cluster randomized trials (RoB 2.0 CRT), and the Risk of Bias in Non-Randomized Studies of Interventions (ROBINS-I) tool by Sterne et al. (2016). The first author and a trained research assistant individually assessed each study for risk of bias. Next, differences were assessed and discussed among the first and second author to determine the final decision.

### Data Preparation for Synthesis

To compare effects across studies, we calculated effect sizes in the form of standardized mean difference scores for all the relevant psychosexual outcomes of the investigated programs (Ellis, 2010). We calculated pooled *d*_*Cohen*_ for pre-posttest designs without a control group (Lenhard & Lenhard, 2016), Morris’ *d*_*ppc2*_ (2008) for designs with a control group, and odds ratios for dichotomous outcomes that both control for baseline scores, using Lenhard and Lenhard’s (2016) effect size calculator. To convert medians and interquartile distances into standardized mean differences, we used DeCoster’s (2009) Excel Macro, and to convert odds ratios, we used the Excel Macro by Wan et al. (2014).

All studies and programs were taken together for synthesis. No pre-specified subgroups were made based on characteristics. We used the rules of thumb for effect sizes based on the new effect size rules of thumb by Sawilowsky (2009), stating that for *d,* 0.01 = very small, 0.2 = small, 0.5 = medium, 0.8 = large, 1.2 = very large and 2.0 = huge. We used *α* = 0.05 as a threshold for determining whether effects were significant. In the presentation of the results, we narratively synthesized these results and investigated potential explanations for heterogeneity among study results, such as whether significant results could be explained by certain program or study characteristics. Next, studies will be indicated with the letter *k* and programs with the letter *p*.

## Results

We included a total of 17 peer-reviewed published papers that reported about 15 different effectiveness studies (i.e., some follow-up measurements of the same study were presented in different papers), of 13 unique programs from seven countries. For an overview, see Table 1.

**Table 1:** Characteristics of the included studies

Program	Study	Design	Time points after end of program	Sample size at baseline (% allocated to intervention)	Retention						Baseline age in years/grade	Country	Ethnic/racial background of the sample
					Post-test			Follow-up					
					Total	Int	Cont	Total	Int	Cont			
Breaking gender barriers	Pulerwitz et al. (2015)	Pre-post	3 months	Students: *N* = 606 Workers: *N* = 549 (100%)	Students: 72% Workers: 39.9% (but new workers filled in post-test survey)						Students: *M* = 17.8 Workers: *M* = 20.3	Republic of China	Chinese
Coaching boys into men (CBIM)	Miller et al. (2012, 2013)	RCT	1. Direct post-test 2. 12 months	*N* = 2,006 (50.2%)	89%	83%	95%	81%	72%	86%	9–12th grade 14–18	USA	American, diverse racial composition
	Miller et al. (2020b)	RCT	1. 3 months 2. 9 months	*N* = 973 (53.2%)	67.8%	70%	65.3%	73.3%	72%	75.1%	*M* = 15.5	USA	American, diverse racial composition
Do Kadam	Santhya et al. (2019), Santhya and Zavier (2022)	QE	1. Direct post-test 2. 5 years	*N* = 1,149 (50.7%)	90%	88.5%	91.2%	74.2%	73.9%	74.6%	*M* = 15.7	India	–
Make a move	Van Lieshout et al. (2019)	RCT	1. Direct post-test 2. 6 months	*N* = 177 (44%)	78.5%	82.%	75.8%	38.4%	28.2%	46.5%	*M* = 14.8	The Netherlands	Mostly Dutch
Male norms initiative	Pulerwitz et al. (2014)	QE	2 months (6 months after baseline, intervention ran for 4 months)	*N* = 729 (33.5% group education + community intervention, 27.2% to comparison)	89%	96.3%	80.3%				Median: 19	Ethiopia	Ethiopian
Manhood 2.0	Miller et al. (2020a)	RCT	1. Direct post-test 2. 3 months 3. 9 months	*N* = 866 (53.7%)	67.8%	70%	65.3%	73.3%	72%	75.1%	*M* = 15.5	USA	Mostly Black American
Parivartan	Miller et al. (2014)	QE	6 months	*N* = 309 (54.4%)	47%						*M* = 12.7	India	–
Program H	Foley et al. (2015)	Pre-post	Direct post-test	*N* = 8 (100%)	87.5%						10–11	USA	Mostly Black American

Program	Study	Design	Time points after baseline	Sample size at baseline (% allocated to intervention)	Retention						Baseline age in years/grade	Country	Ethnic/racial background
					Post-test			Follow-up					
					Total	Int	Cont	Total	Int	Cont			
Program H and Program M	Powell-Williams et al. (2020)	Pre-post	Direct post-test	*N* = 15 (100%)	73.3%						*M* = 10.6	USA	Mostly Black American
Rock and water	De Graaf et al. (2016)	QE	1. Direct post-test 2. 4 to 5 months	*N* = 521 (49.9%)	99.4%	98.5%	100.4%	89.1%	89.6%	88.5%	*M* = 15.2	The Netherlands	Mostly Dutch
Reducing sexism and violence program (RSVP)	Banyard et al. (2019)	QE	Varied: 56 days in control; 72, 84, or 125 days in intervention	*N* = 340 (50%)	85.9%	82.2%	90.3%				*M* = 12.5	USA	Mostly White American
Stepping stones and creating futures	Gibbs et al. (2019)	RCT	1. Direct post-test 2. 12 months	*N* = 674 (50.1%)	64.7%	65.6%	64%	82%	88.1%	82%	*M* = 23.8	South Africa	–
Your moment of truth (YMOT)	Keller et al. (2017)	QE	1. Direct post-test 2. 4.5 months 3. 9 months	*N* = 1,543 (81%)	–	–		85.9%	86.9%	81.6%	*M* = 18.0	Kenya	–
Yuva Samaanta Ki Oor	Freudberg et al. (2018)	Pre-post	12 months	*N* = 137 (100%)	51.1% (purposely sampled 70 participants for post-test)						*M* = 19.00	India	–

RCT = Cluster-Randomized Controlled Trial, QE = Quasi-Experimental Design. Int. = intervention condition, Cont. = control condition

### Study Design

Study designs characteristics are presented in Table 1. Study designs were more or less equally divided over cluster randomized controlled trials (RCT, *k* = 5), semi-controlled evaluation designs (quasi-experimental, *k* = 6) and non-controlled designs (pre-posttest studies, *k* = 4). Eight studies included more than one outcome measurement after the program-period. This was mostly a direct post-test (*k* = 7) or three months after the end of the program (*k* = 1) as the first outcome measurement. For the follow-up measurement(s), intervals ranged from three months up to five years after the post-test. The other studies (*k* = 7) included only one outcome measurement, with intervals ranging from directly (*k* = 2), 2 to 6 months (*k* = 4) up to 12 months (*k* = 1) after the program ended. Hence, the term “pre-posttest study” does not necessarily mean that outcomes were assessed directly post-program. For most (*k* = 10) studies, the final measurement was at least 3 months after the end of the program.

Study sample sizes ranged from *N* = 8 up to *N* = 2,006 participants at baseline, but almost all studies had more than 100 participants in the program at the start of the study (*k* = 13). Retention rates in the intervention groups ranged from 39.9 up to 98.5% at post-test, and 28.2 to 89.6% at follow-up. Of the 14 studies of which we have information about retention at post-test, six had at least a retention rate of 75% at post-test, whereas at follow-up this was the case for *k* = 4 studies (see Table 1).

Regarding assessed outcomes from the perspective of the TPB, the most assessed psychosexual program outcomes in the studies were attitudes (*k* = 13) and behaviors (*k* = 11). Less common were intentions (*k* = 5), perceived behavioral control (*k* = 2). Social norms were assessed in only one study (Van Lieshout et al., 2019).

### Study Context

The context of the included studies is presented in Table 1. Studies were conducted in the United States of America (USA; *k* = 6), India (*k* = 3), African countries (*k* = 3, South-Africa, Kenya, and Ethiopia), the Netherlands (*k* = 2) and Republic of China (*k* = 1). For the six studies conducted in the USA, samples consisted of mostly black participants (*k* = 3), participants of mixed racial composition (*k* = 2) or white American participants (*k* = 1). In most other countries, the population consisted of (mainly) the local majority (*k* = 7), and for two studies, the composition of the sample was not mentioned. The mean age of the studies’ samples ranged between 10 and 23.5 years. Participants were on average between 10 and 13 years (*k* = 4), 15 and 16 years old (*k* = 6) or 18 and 23.5 years (*k* = 5). In *k* = 14 out of 15 studies, participants’ individual maximum age was 24 years. In one study (Gibbs et al., 2020), the individual age range was wider (i.e., 18–38 years) but skewed to the right, resulting in an estimated sample mean age of 23.8 years. Finally, what type of SDV was addressed by the programs as described in the studies, and its definition and operationalization (if any), is presented in Table C1 in Appendix 3.

### Program Characteristics

#### Program Context

The characteristics of the 13 included programs are presented in Table 2. Most programs were implemented in an urban context (*p* = 7), one in a suburban area and the rest of the studies were conducted in rural areas (*p* = 3) or in multiple areas (*p* = 3). For one program this was unknown. About half of the programs were implemented at schools (*p* = 7). Other sites were in the community (*p* = 5), at the workplace (*p* = 1), or in a care setting (i.e., residential youth care, *p* = 1).

**Table 2:** Characteristics of the evaluated programs

Program name	Intended psychosexual outcomes	Number of sessions	Total length in hours	Primary delivery style*	Gender of facilitator	Primary type of facilitator	Target group	Location	Site
Breaking gender barriers	To foster positive/equitable gender norms, as well as provide information on and support the development of skills for HIV/STI and violence prevention	8	16	Lecture/presentation	–	Workers: trained facilitators Students: teacher	1. Factory workers 2. Vocational students	Multiple areas	School and workplace
CBIM	To prevent gender-based violence and alter norms that foster violence perpetration	11	1.83–2.75	Interactive presentation	Male	Coaches	Athletic teams	Urban/multiple areas	School
Do Kadam	To promote egalitarian gender attitudes and rejection of violence against women and girls	42–48	42–48	Active participation	Male	Peer facilitators	Unmarried boys who were members of youth clubs	Rural	Community
Make a move	To prevent sexual harassment and to promote respectful (sexual) relationships	8	12	Active participation	Male and female co-facilitators, or male, or female	Professionals	Boys in residential youth care	Multiple areas	Care
Male norms initiative	To promote critical reflection on and identify the negative outcomes of common gender norms that increase the risk of violence and the potential positive aspects of more gender-equitable behavior, and to engage the wider community in supporting a shift in specific harmful norms	8	16–24	Active participation	Male	Peer facilitators	Young men	Suburban	Community
Manhood 2.0	To prevent SDV, challenge homophobia, harassment, and harmful masculinity norms, and to demonstrate more respectful behaviors combined with bystander and healthy sexuality skills	6	3	Active participation	–	Professionals	Young men from disadvantaged neighborhoods	Urban	Community
Parivartan	To increase positive bystander behaviors and improve gender attitudes	12	9–12	Lecture/presentation	Male	Coaches	Cricket athletes	Urban	School
Program H	To reduce attitudes conductive to gender-based violence	7–10	–/10	Interactive presentation	–/Two male teachers + rape crisis centre employee	Teacher and professional	Students at risk of not reaching/maintaining academic grade level performance	Urban	Elementary school
Rock and water	To prevent sexually aggressive behavior and sexual aggression-supportive cognitions	7 or 10	10–10.5	Active participation	–	Teacher	Boys and male adolescents	Unknown	School
RSVP	To prevent SDV and explore the normalization, pervasiveness, and harmful nature of rigid gender role assumptions	4	4	Active participation	–	Professionals	Middle school boys	Rural	School
Stepping stones and creating futures	To reduce intimate partner violence and HIV risk, promote gender equity	21	63	Active participation	–	Professionals	Young people not formally employed or enrolled in education	Urban	Community
YMOT	To change attitudes and intervention behaviors related to gender-based violence	6 + 2 refresher sessions	16	Active participation	Male	Professionals	Boys living in the slums of Nairobi	Urban	School
Yuva Samaanta Ki Oor	To change individual and community level knowledge, attitudes, and behaviors regarding gender, violence, and sexuality, and increase participation in developing and sustaining gender equal social norms in communities	26	Unknown	Active participation	Male	Group leader of community, elected or assigned	Young adults in communities with high levels of intimate partner violence	Rural	Community

Primary delivery style is divided into three categories that build up in activity of the participants: (1) Lecture/presentation (i.e., passive participants, mostly listening); (2) Interactive presentation (i.e., participants listening actively, with some discussion, questions etc.); (3) Active participants (e.g., participant role plays, participant discussion groups, projects, theatre plays performed by the participants)

#### Intended Psychosexual Outcomes

In about half the programs (*p* = 6) the main intended psychosexual outcome was to prevent SDV behaviors along with the attitudes of social norms conductive to SDV. For four programs, the main psychosexual outcome was changing attitudes related to SDV (*p* = 2), or changing attitudes and promoting SDV bystander behaviors (*p* = 2). Finally, for two programs the intended psychosexual outcomes were norms (*p* = 1) or norms and skills necessary for SDV prevention (*p* = 1).

#### Target Group

The programs were designed for various specific target groups of male youth, but the participants were often selected for the program based on some indicated risk factor for encountering or perpetrating SDV, including: (1) age (e.g., elementary school age, *p* = 3); (2) living situation (e.g., disadvantaged neighborhood; *p* = 4), (3) culture (e.g., country or community with high rates of intimate partner violence; *p* = 2), or (4) activity/employment status (e.g., being currently unemployed; *p* = 4) (see Table 2).

#### Facilitators

Program facilitators were most often professionals (e.g., adult employees of the program evaluation project or adults who received extensive training in sexual violence prevention and/or the program model, *p* = 5), see Table 2. Other facilitators were peer facilitators (i.e., youth of similar age and gender who were trained by the program staff, *p* = 3), or people who received some training in the program manual but were not professionals in SDV prevention, such as teachers or coaches (*p* = 3). Two programs were delivered by a combination of a professional (e.g., an employee of a rape crisis center), and someone who may have received some training to implement the program (e.g., a teacher), simultaneously (*p* = 2). When the gender of the facilitator was mentioned, they were most often male (*p* = 8) and for one program, a female (co-)trainer could also facilitate the program. Notably, for the other five programs, facilitator gender was not reported.

#### Duration and Intensity

Programs consisted of a broad range of sessions (varying from four to 48 sessions), see Table 2. Except for *p* = 3 programs, which each had over 20 sessions, all programs (*p* = 10) consisted of less than or around 10 sessions. In terms of intensity (i.e., total duration), programs ranged from less than 2 up to 96 h. In terms of frequency, most programs had weekly sessions. Session length could range from 10 to 15 min, up to 3 h per session.

#### Work Forms

Most programs included various work forms (lectures/presentations, discussions, role plays), and also actively involved the participants, see Table 2. This included discussions among participants, role plays and other activities where participants are stimulated to work on acquiring new knowledge, skills and critically reflect on behavior and norms. In about one third of the programs (*p* = 4), facilitators were more active than participants, as they presented the program mostly in the form of interactive presentations or lectures. In the other programs, participants were most active (e.g., discussions, role plays).

### Program Content

From the published papers, we identified 12 different categories of topics that were discussed or taught within the programs, which are presented in Table 3. The most common topic was gender, which was mentioned for *p* = 12 programs. For most programs (*p* = 9), but not all, sexuality was mentioned as a program topic. For one program (Program H), the authors mentioned that leaving sexuality out of the program was a deliberate choice due to the young target group of about 10 years (Program H; Foley et al., 2015; Powell-Williams et al., 2020). Hence, they focused more on emotions and gender than on sexuality. Norms and skills were both mentioned for *p* = 9 programs. Notably, masculinity as a specific topic, and defensibility against SDV were both mentioned for only *p* = 3 programs.

**Table 3:** Topics discussed in the programs as reported in the studies

Program (Country)	Gender	Violence	Sexuality	Norms	Skills	Relationships	Respect	Sexual consent	Emotions	Communication	Masculinity	SDV defensibility
Breaking gender barriers (China)	V	V	V	V	V	V						
CBIM (USA)	V	V	V	V	V		V				V	
Do Kadam (India)	V	V	V	V		V	V	V	V			
Make a move (the Netherlands)	V		V	V	V	V	V	V		V	V	V
Male norms initiative (Ethiopia)	V	V	V	V								
Manhood 2.0 (USA)	V		V	V		V	V					
Parivartan (India)	V	V		V	V		V					
Program H (adapted) (USA)	V	V		V	V		V		V	V	V	
Rock & Water (the Netherlands)		V	V		V		V	V	V	V		V
RSVP-MSP (USA)	V	V		V	V	V			V			
Stepping stones and creating futures (South Africa)	V	V	V		V	V						
Your moment of truth (Kenya)	V	V			V	V		V		V		V
Yuva Samaanta Ki Oor (India)	V	V	V									
Discussed in (*p*/13) programs	12	11	9	9	9	7	7	4	4	4	3	3

### Program Integrity

About half of the studies reported information on program integrity during the study (*k* = 7). Information about the dosage (*k* = 5) and/or adherence (*k* = 4) was available either directly from the papers (*k* = 4), or upon request via personal communication with the authors (*k* = 2), or not, despite several reminders to send the requested information (*k* = 3). The reported dosage ranged from 33.7 up to 98.8%, and the reported adherence ranged from 54 up to 99.5%.

### Program Effectiveness

In total, we retrieved 121 effect sizes: 61 at post-test and 60 at follow-up. Out of the 121 effect sizes, a total of 37 (30.6%) were significant (18 at post-test and 19 at follow-up). All significant effects were in the hypothesized direction. Measurement intervals at post-test ranged from directly—12 months after the program had ended. For the follow-up, this was 3 months up to 5 years after the posttest. Hence, the studies represented a broad range of short-term and longer-term effects (See Fig. 1).

Importantly, two studies investigating Program H (Foley et al., 2015, and Powell-Williams et al., 2020), only presented their results and analyses on item-level. It was also not possible to retrieve effectiveness information for the total scales of interest from the authors. As a result, these two studies were excluded from this part of the results section, resulting in 13 studies about 12 programs for which effect sizes are reported and described below. They are grouped according to the elements of the TPB (i.e., behaviors, intentions, attitudes, social norms and perceived behavioral control).

For almost all programs (*p* = 12), significant effects on at least one assessed outcome were found, only one program showed no significant effects whatsoever. At post-test (directly up to 12 months after the program ended), most effect sizes were (very) small (61.1%). At follow-up (3 months–5 years after the post-test), most significant effects were also (very) small (63.1%), but another 31.6% were large/huge. A summary of the proportion of significant effects (post-test and follow-up) per TPB-factor are presented in Fig. 2.

#### Effectiveness on Behaviors

Almost all included studies assessed behavioral outcomes (*k* = 11; 52 effect sizes; see Fig. 1 and Table 4). The assessed behavior-related outcomes could be divided into two categories: SDV perpetration (e.g., making sexual comments, sexual coercion) and bystander behaviors (e.g., intervening when witnessing sexual harassment, or laughing). Notably, none of the studies assessed SDV victimization as an outcome. In total, 20 significant effects (38.5%) were reported. One of the 52 effect sizes was a result of a composite measure of various behaviors related to gender, sexuality, and violence, which showed a small effect at post-test.

**Table 4:** Outcomes of the programs

Program	Study	Outcome(s)	Pre-post effect	Intervention effect		Sample size					
						Post-test			Follow-up		
			Post-test	Post-test	Follow-up	Total	*n*_intervention_	*n*_control_	Total	*n*_intervention_	*n*_control_
*Behavior*											
Breaking gender barriers	Pulerwitz et al. (2015)^a^	Either physical or verbal violence towards a non-partner female	W: 0.11			219	219				
			S: − 0.64*			496	496				
		Either physical or verbal violence towards a female partner	W: − 0.55*			219	219				
			S: − 0.71*			496	496				
CBIM	Miller et al. (2012, 2013)	DV perpetration among those who have dated		− 0.10	− 0.20*	1754	829	925	1194	537	657
		Any dating or cyber-SV, or sexual harassment		0.13	0.05	920	491	429	824	428	396
		DV perpetration among those who have dated		0.06	− 0.03*	920	491	429	824	428	396
Do Kadam	Santhya and Zavier (2022)	Emotional, physical, or sexual violence against an intimate partner		0.66		853	431	422			
		Perpetration of physical or sexual violence against an intimate partner in household		0.634		853	431	422			
Male norms initiative	Pulerwitz et al. (2014)^b, c^	Any type of intimate partner violence perpetration (physical, sexual, or psychological)		0.02		486	251	235			
Manhood 2.0	Miller et al. (2020a)^b^	Any SV towards a dating- or non-dating partner			0.16	587	325	262	635	334	301
		Any relationship violence			− 0.47						
Parivartan	Miller et al. (2014)	Any SV perpetration towards a female			− 0.03				309	168	141
		Any dating violence perpetration			0.09	587	325	262	635	334	301
Rock and water	De Graaf et al. (2016)	Sexual aggression pressure methods (i.e., using verbal manipulation, coercion or taking advantage of a situation)			− 0.23*				455	229	226
		Non-physical sexual aggression (online or in real life)			− 0.16				455	229	226
Steppings stones and creating futures	Gibbs et al. (2019)	Past year physical IPV		− 0.17	− 0.18*	440	224	216	505	237	268
		Past year sexual IPV		− 0.22	− 0.16*	440	224	216	505	237	268
		Severe past year IPV		− 0.14	− 0.19*	440	224	216	505	237	268
		Controlling behaviors over primary partner		− 0.15***	0	440	224	216	505	237	268
		Emotional IPV		− 0.13	− 0.15	440	224	216	505	237	268
		Economic IPV		− 0.15	− 0.20*	440	224	216	505	237	268
		Non-partner SV		− 0.09	− 0.19*	440	224	216	505	237	268
CBIM	Miller et al. (2012, 2013)	Positive Bystander intervention^d^		0.18*	0.01	1,754	829	925	1194	537	657
		Negative Bystander behavior^b^		− 0.02	− 0.03*	1,754	829	925	1194	537	657
	Miller et al. (2020b)	Positive bystander intervention			4.34*				824	428	396
		Negative bystander behavior^b^			− 0.83				824	428	396
Do Kadam	Santhya et al. (2019), Santhya and Zavier (2022)	Bystander intervention with verbal or physical violence in household			0.08	853	431	422			
		Bystander intervention with verbal, physical or sexual violence in their community		0.36*	0.02	853	431	422	853	431	422
Manhood 2.0	Miller et al. (2020a)^b^	Positive bystander intervention^d^		0.00	0.00						
		Negative bystander behavior^b^		0.00	0.00						
Parivartan	Miller et al. (2014)	Positive bystander intervention^d^			0.01				309	168	141
		Negative bystander behavior^b^			− 0.22						
YMOT	Keller et al. (2017)	Successfully intervened when witnessing verbal harassment			0.82***				1325	1086	239
		Successfully intervened when witnessing physical threatening			0.77***				1325	1086	239
		Successfully intervened when witnessing physical sexual assault			1.18***				1325	1086	239
Yuva Samaanta Ki Oor	Freudberg et al. (2018)	Composite measure: Behavior related to gender, violence, and sexuality	0.38*			70	70				
*Intentions*											
CBIM	Miller et al. (2012, 2013)	Intention to intervene		0.20*	0.11	1754	829	925	1194	537	657
	Miller et al. (2020b)	Intention to intervene		− 0.25	− 0.98	920	491	429	824	428	396
Manhood 2.0	Miller et al. (2020a)	Intention to intervene		1.82	3.50*	587	325	262	635	334	301
Parivartan	Miller et al. (2014)	Intention to intervene			0.25				309	168	141
RSVP	Banyard et al. (2019)	Helping intentions		− 0.07		144	72	72			
Make a Move	Van Lieshout et al. (2019)	Intentions to whine, persuade or get angry if a partner does not want to have sex		− 0.01	0.01	123	64	59	68	22	46
*Attitudes*											
YMOT	Keller et al. (2017)	Positive Women Composite (attitude towards women and rape myths)			2.01***				1325	1086	239
Yuva Samaanta Ki Oor	Freudberg et al. (2018)	Attitudes regarding gender, sexuality, and violence	1.86***			70	70				
Breaking gender barriers	Pulerwitz et al. (2015)	Gender equitable men scale	W: 0.25***			219	219				
			S: 0.12*			496	496				
CBIM	Miller et al. (2012, 2013)	Gender equitable attitudes		0.00	− 0.05	1754	829	925	1194	537	657
	Miller et al. (2020b)	Gender equitable attitudes		0.67	1.78	920	491	429	824	428	396
Do Kadam	Santhya and Zavier (2022)	Gender equitable attitudes and notions of positive masculinity		0.20**	0.10*	853	431	422	853	431	422
Male Norms Initiative	Pulerwitz et al. (2014)^c^	Gender equitable men scale		0.14**		486	251	235			
Make a Move	Van Lieshout et al. (2019)	Adversarial sexual beliefs		0.01	− 0.06	123	64	59	68	22	46
Manhood 2.0	Miller et al. (2020a)	Gender equitable attitudes		0.00	− 0.25	587	325	262	635	334	301
Parivartan	Miller et al. (2014)	Gender equitable attitudes			0.37***				309	168	141
RSVP	Banyard et al. (2019)	Adherence with traditional masculine gender norms		− 0.02		144	72	72			
		Support for gender equity in relationships		0.40		144	72	72			
Stepping stones and creating futures	Gibbs et al. (2019)	Gender equitable attitudes		0.65***	0.03	440	224	216	505	237	268
CBIM	Miller et al. (2012, 2013)	Recognition of abusive behavior		0.06	− 0.02	1754	829	925	1194	537	657
	Miller et al. (2020b)	Recognition of abusive behavior		0.94*	2.41*	920	491	429	824	428	396
Do Kadam	Santhya et al. (2019), Santhya and Zavier (2022)	Attitudes rejecting men's controlling behaviors over sister, wife, or girlfriend		0.24***	0.12	853	431	422	853	431	422
		Attitudes rejecting violence against women and girls		0.29***	0.00	853	431	422	853	431	422
Make a Move	Van Lieshout et al. (2019)	Attitude towards communication about sexual wishes and boundaries		0.04	− 0.01	123	64	59	68	22	46
		Attitude towards sexual self-control		0.17	0.27	123	64	59	68	22	46
		Attitude towards dating violence		0.09	− 0.19	123	64	59	68	22	46
		Rape attitude		0.07	0.24	123	64	59	68	22	46
Manhood 2.0	Miller et al. (2020a)	Recognition of abusive behavior		− 0.56	2.38	587	325	262	635	334	301
Parivartan	Miller et al. (2014)	Attitudes disapproving of violence against women			0.04				309	168	141
Rock and Water	De Graaf et al. (2016)	Attitudes towards sexual pressure/dating violence		− 0.24	− 0.47	517	256	261	455	229	226
RSVP	Banyard et al. (2019)	Agreement with norms for violence prevention^e^		− 0.07		144	72	72			
		Support for male power		− 0.21		144	72	72			
		Support for male violence		− 0.36		144	72	72			
*Social norms*											
Make a Move	Van Lieshout et al. (2019)	Social norms: friends’ acceptance of DV		0.38	0.46*	123	64	59	68	22	46
*Perceived behavioral control*											
Make a move	Van Lieshout et al. (2019)	Self-efficacy: sexual self-control		0.11	− 0.80	123	64	59	68	22	46
		Self-efficacy: communication about sexual wishes and boundaries		0.35	0.28	123	64	59	68	22	46
		Outcome expectancies of persuading a partner into having sex		0.22	− 0.20	123	64	59	68	22	46
Rock and water	De Graaf et al. (2016)	Experienced control during sexual interactions		− 0.27	0.13	517	256	261	455	229	226
		Assertiveness of boy’s own sexual experiences		− 0.17	0.00	517	256	261	455	229	226

Effects are presented for pre-posttest studies (conducting within-group analyses) and (quasi)-experimental studies which conducted between-group analyses. The post-test period for Breaking Gender Barriers was 3 months, for Yuva Samaanta Ki Oor 12 months after the program ended

^a^In Pulerwitz et al. (2015), two different samples were investigated (factory workers and vocational education students). W stands for workers and S for students

^b^For SDV perpetration, within-group over time effects were also reported for Manhood 2.0 and Male Norms Initiative, and here significant reductions in perpetration were found for the intervention group at follow-up. Within-group over time effects were also reported and significant for Manhood 2.0 on reduced negative bystander behaviors at post-test and follow-up, and recognition of abusive behavior significantly increased over time to post-test and to follow-up

^c^We used the comparison of the group education + community intervention and comparison group, because tests were reported for this comparison

^d^Positive bystander intervention is disrupting the SDV behaviors, saying something about it or talking to an adult. Negative bystander behavior is supporting, laughing, or not doing anything

^e^We coded the outcome ‘injunctive norms regarding violence prevention’ as ‘attitudes’ because the used instrument seemed to assess participants’ own attitudes, not the perceived attitudes of others

*p < .05; **p < .01; ***p ≤ .001

Regarding SDV perpetration, 33 effect sizes were retrieved. Twelve effects (36.4%) were significant. Regarding bystander behaviors, seven effects (38.9%) were significant. For SDV perpetration, all significant effects were (very) small. For bystander behaviors, effects were larger. Concluding, more evidence for effectiveness on behavior was found at follow-up (65%) than at direct post-test. Most significant effects were (very) small.

#### Effectiveness on Intentions

Six studies investigated intentions (10 effect sizes see Fig. 1 and Table 4). Two categories of intentions were found: intentions for perpetration of SDV and intentions to intervene (i.e., helping/bystander intentions). Two (25%) of the effects on bystander intentions were significant, and the effects were larger at follow-up than at post-test, whereas no evidence for effectiveness on intentions for perpetration of SDV was found.

#### Effectiveness on Attitudes

All studies assessed program effects on attitudes (*k* = 13; 44 effect sizes, see Fig. 1 and Table 4). The outcomes that were measured regarding attitudes related to SDV could be divided into two categories: attitudes towards SDV and gender equitable attitudes. In total, 13 effects were significant (31.8%).

Regarding attitudes towards SDV, 24 effect sizes were included. Four effects (16.7%) were significant. Regarding gender equitable attitudes, 18 effect sizes were included. Seven of the effect sizes were significant (39%) The effects were mostly small. Two effect sizes were reported for attitudes as one composite outcome, and these showed very large effects at post-test and follow-up. Concluding, more evidence for effectiveness was found for changing gender equitable attitudes than attitudes towards SDV. For both categories, effects were mostly small, and most effects were found at post-test.

#### Effectiveness on Social Norms

Social norms (friends’ acceptance of SDV) were assessed in one study (Van Lieshout et al., 2019), with two effect sizes (*k* = 1; see Fig. 1 and Table 4). Van Lieshout et al. (2019) found one small effect at follow-up, *d* = 0.46. While the effect had a *p*-value of *p* = 0.03, the authors of this paper did not consider this a significant result, as they corrected for multiple testing using *α* = 0.005.

#### Effectiveness on Perceived Behavioral Control

Outcomes related to perceived behavioral control (see Fig. 1 and Table 4) were assessed in two studies resulting in 10 included effect sizes (*k* = 2). Examples of outcomes that were assessed regarding this concept are outcome expectancies of persuading a partner into having sex (four effects, Van Lieshout et al., 2019), and experienced assertiveness during sexual experiences (six effects, De Graaf et al., 2016). None of the effects were significant.

### Risk of Bias in the Included Studies

The risk of bias assessment is included in Table 5. All included studies presented some problems with risk of bias. Of the *k* = 5 RCTs, *k* = 2 were judged as having some concerns for risk of bias, and *k* = 3 were judged as high risk, with problems arising from three main domains. The first was allocation concealment: it was often unclear whether allocation of the cluster could have influenced participant selection (all studies were judged as some concerns). The second concern was lack of blinding in all studies, as participants may therefore have responded differently. The third was incompleteness of outcome data: attrition might have been related to the true outcome which possibly leads to overestimating effects, or missing data were not properly addressed (*k* = 3 were judged as high risk). It should be mentioned that bias due to attrition does not necessarily lower quality of a study but may nevertheless present a risk of bias in the estimation of effects.

**Table 5:** Risk of bias

Authors (year)	Design	Sequence generation	Allocation concealment	Blinding	Incomplete outcome data		Selective outcome reporting	Measurement of the outcome	Overall risk of bias
*Randomized controlled trials assessed with the RoB-2 tool*									
Van Lieshout et al. (2019)	RCT	Low risk	Some concerns	Some concerns	High risk		High risk	High risk	High
Miller et al. (2012, 2013)	RCT	Low risk	Some concerns	Some concerns	High risk		Low risk	Some concerns	High
Miller et al. (2020a)	RCT	Low risk	Some concerns	Some concerns	High risk		Low risk	Some concerns	High
Miller et al. (2020b)	RCT	Low risk	Some concerns	Some concerns	Low risk		Low risk	Some concerns	Some concerns
Gibbs et al. (2019)	RCT	Low risk	Some concerns	Some concerns	Low risk		Low risk	Some concerns	Some concerns

	Design	Confounding	Classification in interventions	Selection of participants	Incomplete outcome data	Deviations from intended intervention	Selective outcome reporting	Measurement of the outcome	Overall risk of bias
*Non-randomized trials assessed with the ROBINS-I tool*									
Banyard et al. (2019)	QE	Low	Low	Low	Moderate	NI	Moderate	Serious	Serious
De Graaf et al. (2016)	QE	Moderate	Low	Moderate	Low	Serious	Moderate	Moderate	Moderate
Freudberg et al. (2018)	Pre-post	Moderate	Low	Serious	Serious	Low	Serious	Serious	Serious
Keller et al. (2017)	QE	Serious	Low	Low	Serious	NI	Serious	Moderate	Serious
Miller et al. (2014)	QE	Moderate	Low	Moderate	Moderate	Low	Moderate	Moderate	Moderate
Pulerwitz et al. (2014)	QE	Moderate	Low	Moderate	Low	NI	Moderate	Moderate	Moderate
Pulerwitz et al. (2015)	Pre-post	Moderate	Low	Moderate	Low	Low	Moderate	Moderate	Moderate
Santhya et al. (2019), Santhya and Zavier (2022)	QE	Moderate	Low	Low	Low	Low	Moderate	Moderate	Moderate

Regarding the *k* = 8 non-randomized studies, *k* = 5 studies were judged as moderate, and *k* = 3 as serious risk, arising from problems on three main domains (Table 5). The first was confounding, as possibility, effects of the program could have been due to another factor than program participation. Authors did not control for this using appropriate measures or used measures with unknown or poor validity/reliability (*k* = 6 were judged as moderate, *k* = 1 as serious risk). The second was measurement of the outcome, as self-report in combination with knowledge of intervention status may have biased participants’ answers (*k* = 4 were judged as moderate and *k* = 2 as serious risk). The third domain was incomplete outcome data, relating to problems with adhering to reporting standards or selective outcome reporting, which was often not possible to assess because of lack of pre-registration (*k* = 2 were judged as moderate and *k* = 2 judged as serious risk). In sum, the assessment of effectiveness of the studies included in this review was based on five RCTs and eight non-randomized studies, all with moderate to serious risk of bias.

## Discussion

Sexual and dating violence (SDV) among youth is a worldwide problem, and male youth are specifically at risk of perpetrating SDV. We systematically reviewed 15 studies, from seven countries, evaluating 13 different programs to get insight into the form and content, intended psychosexual outcomes, and effectiveness of SDV prevention programs for male youth. Specifically, we looked at programs targeting theory-based underlying risk factors for SDV such as attitudes, social norms, (perceived) behavioral control (e.g., skills) and intentions (according to the TPB; Li et al., 2010; Miller, 2010), that are multi-session, group-focused and interaction-based.

### Characteristics of the Programs

#### Location, Facilitators, Duration and Intensity

Regarding program characteristics, two things stood out concerning location and facilitators, and duration and intensity. First, in lower income countries, programs were more often situated in the community and facilitators were mostly peers or community leaders (India and Ethiopia), whereas in higher-income countries (USA and the Netherlands), programs were often implemented at schools and facilitators were typically professionals or teachers. Moreover, facilitators were mostly male, but the facilitator’s gender was often not clear. Currently the possible effect of facilitator gender on program effectiveness remains therefore unknown. Second, duration and intensity differed widely. However, unlike other reviews (e.g., DeGue et al., 2014), we did not find that higher intensity was related to more program effectiveness. Instead, it could be that duration is a more important factor: one of the most effective programs (assessed three times, Miller et al., 2012, 2013, 2020b) lasted only two hours in total but was stretched over a longer period of time. Longer durations may enable participants to better internalize and generalize program changed attitudes, and skills in one location to other contexts, such as when with friends and family (Garzón-Orjuela et al., 2021).

#### Program Content

The currently reviewed programs seem to adhere to standards of effective sex and relationships programs—as being comprehensive, skills-based, and addressing social pressures—(UNFPA, 2003), as the most discussed program topics were gender, violence, sexuality, norms, and skills. Moreover, the strong embedding of gender and violence in the curricula is promising, as the gendered nature of SDV as -generally- an act of violence of men against women, is not always embedded in SDV prevention programs (Reed et al., 2010). However, attitude-related topics such as masculinity, and perceived behavioral control factors such as how to obtain sexual consent, and communication, as well as defensibility against SDV were part of less than one third of the programs. Harmful attitudes regarding masculinity -such as being sexually promiscuous, emotionally stoic, homophobic and aggressive (Banyard et al., 2019)- have been consistently linked to SDV perpetration in both male youth and adults (Baugher & Gazmararian, 2015; Taquette & Monteiro, 2019) above the effects of gender equitable attitudes (Banyard et al., 2019; Reidy et al., 2014). Moreover, the absence of discussion of sexual consent may be explained by a focus on prevention of dating violence in general, where sexual interaction may receive less attention. Nevertheless, understanding consent (i.e., to be freely given, reversible, informed, enthusiastic, and specific; Lawder, 2018), may aid in developing the relevant interaction skills needed to prevent SDV (Williams et al., 2022).

Finally, contrasting mixed gender programs (Lee & Wong, 2020; Russel et al., 2021), in the programs for male youth there is a strong focus on the perpetration side of SDV, whereas they can also experience detrimental effects from SDV victimization (Coker et al., 2000; Sears & Byers, 2010). Moreover, the victim-offender overlap (Jennings et al., 2012), and numerous studies finding that youth perpetrating SDV may simultaneously be victims (De Bruijn et al., 2006; Rubio-Garay et al., 2017), as well as the possibility of same-sex victimization, all challenge the heteronormative idea that the prevention of SDV victimization should be solely geared towards females, and of SDV perpetration solely towards males (DeKeseredey et al., 2017; Rollè et al., 2018). Moreover, when male youth are taught to think about, recognize and indicate their own sexual wishes and boundaries, they may also be better at respecting those of others (Laan et al., 2021; Schneider & Hirsch, 2020; Williams et al., 2022).

### Program Effectiveness

Regarding effectiveness on specific outcomes related to the TPB, the fact that most significant effects were found on behaviors, compared to a systematic review on programs also for adult men not being able to find this (DeGue et al., 2014; Wright et al., 2020) suggests that programs focused on male youth may indeed be promising. Another explanation may be that behaviors were mostly effective longer-term, and many of the studies in the current review had relatively long follow-up times to show them. Regarding intentions, we found only limited significant effects and only on bystander intentions. Next to behaviors, most significant effects were found on attitudes. This is not surprising, as attitudes have been found to be changed in many similar meta-analyses and reviews (Anderson & Whiston., 2005; Edwards & Hinsz, 2014; Lee & Wong, 2020; Ting, 2009; Wright et al., 2020). However, attitude change was mostly visible at short-term (69.2% of effects), and less at longer-term (31.8%), suggesting that effects on attitudes may diminish fairly quickly (Anderson & Whiston, 2005). Little evidence was found for program effectiveness on social norms. Moreover, only two studies assessed perceived behavioral control using questions about (fictional) experiences with SDV and found no significant effects. One explanation may be that youth find it difficult to report about situations of SDV and their own (perceived) behavioral control in these situations, especially when they are not yet sexually experienced. Another explanation for the lack of findings might be that the use of role-plays as an important component of these programs, are not real-time enough to be effective (Jouriles et al., 2009).

Overarching, we found two general indicators of effectiveness in the studies. First, we saw that effectiveness studies’ measured outcomes did not always match the programs’ intended psychosexual outcomes. However, when they did, studies were more likely to show effectiveness (for instance see Gibbs et al., 2019; Keller et al., 2017). Importantly, failing to find significant outcomes might be indicative of poor program effectiveness, but can also indicate inappropriate assessment. The second relates to the cultural setting of the programs. While studies from higher income countries (i.e., USA and the Netherlands) accounted for over half of the total assessed effects, only one fifth of these effects were significant, whereas the studies from India and Africa found effectiveness for over half of their assessed outcomes. This suggests that there may be more to be gained in terms of prevention in lower income countries, where rates of SDV are generally also higher (Abrahams et al., 2014; Borumandnia et al., 2020; WHO, 2021). Another explanation may be that most programs implemented in Africa and Asia focused not only on individual participants, but also on bringing community-level changes in SDV related attitudes and behaviors, which has previously been suggested as a promising strategy for attaining effectiveness in SDV prevention (Casey & Lindhorst, 2009). As has also been suggested by other authors, implementing prevention at various structural levels (e.g., not only at school but also in the community) may exacerbate effects (Casey & Lindhorst, 2009; Ruane-McAteer et al., 2020). This may also be the case in higher income countries, because even though these countries might be more individualistic, youth still develop their sexual attitudes and behavioral patterns through their social contexts (De Bruijn et al., 2006; Endendijk et al., 2022; Van de Bongardt et al., 2015).

Regarding overall effectiveness found in the current review, the eight included studies with the largest samples (*N* > 500) accounted for most significant effects (32, 86.5%). It should be noted that most of these significant effects were (very) small (21, 65.6%). However, even small effects on behavior can make meaningful differences, depending on the severity of the behaviors prevented by a program, and its cost-efficiency and scalability (Funder & Ozer, 2019; Kraft, 2020). For instance, relatively small effects were found for Coaching Boys into Men on SDV perpetration, *d* = 0.03 to 0.20 (Miller et al., 2012, 2013, 2020a, 2020b). Nevertheless, the researchers estimated that the relatively cost-efficient program prevented 85 incidents of dating abuse, 48 incidents of sexual harassment and 20 incidents of sexual assault per 1000 participants (Jones et al., 2021). With this result, they estimated a $2.4 million reduction in costs for society, given victim’s long-term health consequences and lost work. To our knowledge, there are currently no guidelines available as to what effect sizes can be considered meaningful for SDV-related concepts. These would surely advance our understanding of how to evaluate SDV prevention program effects, for which numerous previous researchers have also called attention (Breitenbecher, 2000; DeGue et al., 2014; Schewe & O’Donohue, 1993).

Second, the participants in programs that showed much effectiveness (i.e., effective on almost all outcomes) were generally a bit older (between 17 and 24 years old, compared to youth 16 aged or younger). However, most of the studies on these programs also included larger samples and/or had better fit between the intended and assessed outcomes. Moreover, some of these programs (but not all) had relatively many program sessions (i.e., 20 or more compared to less than or around 10). This makes it difficult to say much about what caused the found effects. Regarding other program—or study characteristics, we did not find any clear patterns.

### Suggestions for Practice

From this systematic review, we have three suggestions for future practice in SDV prevention for male youth. First, developers of these programs should take into account the theoretically relevant factors related to SDV behaviors, and in the program curricula more attention should be paid to evidence-based topics such as attitudes regarding masculinity, and skills necessary for (perceived) behavioral control (e.g., how to obtain sexual consent, and defensibility against SDV experiences). Second, online SDV is increasingly on the rise. Examples of online SDV are online grooming, a sequence of behaviors employed by an offender in order to make the victim less resistant to sexual abuse (Sheldon & Howitt, 2007), and the forwarding of a partner’s private nude photos to others without their consent. Male youth are again at risk of perpetrating this type of violence, as well as becoming victims, with the number of experiences and impact of the negative outcomes similar to those of women (Champion et al., 2022). Moreover, studies have indicated that these experiences also potentially have major impact on the victims, as the use of technology increases a perpetrator’s access to and control over the victim (Say et al., 2015; Whittle et al., 2013; Zweig et al., 2013). For a review on the prevention of online SDV, see Ojeda and Del Rey (2022). Third, the lack of significant effects on perceived behavioral control outcomes suggests that program developers may have to look at effective methods of teaching sexual and relationship competence and skills beyond role-plays and discussions. For instance, in research on general aggressive behavior in male youth, there is an upcoming use of VR methods to let participants safely practice in almost real-life aggression-invoking situations (Alsem et al., 2021). Finally, whilst the use and effectiveness of in-person SDV prevention programs have been widely investigated, SDV prevention programs with a digital set-up are on the rise, with promising advantages in terms of cost-efficiency, accessibility, and scalability (Andrade et al., 2022). Moreover, they can be personalized to, for instance, each youth’s dating experiences and subsequent SDV risk profile (Levesque et al., 2016).

### Suggestions for Research

Whilst some studies were well-executed, there was a large variety in study quality, and risk of bias poses a significant problem in determining effectiveness for individual programs, and this type of program in general. Thus, we discuss five suggestions for future research. First, when the match between effectiveness study outcomes and programs’ intended psychosexual outcomes was high, we saw that studies consecutively found better effectiveness. Researchers should thus carefully determine which outcomes are relevant to evaluate and operationalize the outcomes to match the intended psychosexual outcomes of the program. Relatedly, five included programs’ intended psychosexual outcomes were to change norms conductive to SDV and five to change attitudes. Yet only one study evaluated social norms, whereas all studies evaluated attitudes. Social norms can significantly contribute to the perpetration of SDV (Jewkes et al., 2015), In fact, research testing TPB models on sexual behavior (including harassment), consistently found stronger evidence for effects of social norms and perceived behavioral control on intentions/behaviors, and the weakest (or no) evidence for attitudes (Li et al., 2010; Lin et al., 2021b; Simms & Byers, 2013). Changing social norms is a unique asset of group focused and interaction-based programs (Berkowitz, 2010). Moreover, perceived behavioral control (for instance, in terms of communication skills), is crucial for both positive as well as negative SDV-related behaviors to show (Ajzen, 1991; Lin et al., 2021a, 2021b). Hence besides attitudes, more attention could be paid to the other theoretically relevant antecedents of SDV. Second, assessing and reporting program integrity is highly important for determining effectiveness (Bellg et al, 2004; Perepletchikova & Kazdin, 2005). We found that dosage can be as low as one third, and adherence as low as 50%, and one study found that when adjusting for the minimum dosage required for program effectiveness, effects on one outcome changed from non-significant to significant (Miller et al., 2012). Third, as most programs were evaluated only once, studies should be designed to evaluate programs multiple times using dynamic logic modelling (Ruane-McAteer et al., 2020). In doing so, studies should take ample follow-up time as well as oversample, as behaviors showed lagged effects, and retention rates at follow-up were generally low. Suggested follow-up time is one year (Ricardo et al., 2011), but future research may also further investigate when SDV-related factors become stable to inform meaningful follow-up times. Especially when researchers are limited in resources to use rigorous evaluation designs, they should try to diminish attrition, and properly investigate effects of attrition on the outcome (Bellg et al., 2004). Fourth, in our review process, we noticed that programs are generally described in quite limited detail in published papers, a common problem (Michie et al., 2009). Proper program description using the Template for Intervention Description and Replication guidelines (TIDieR; Hoffmann et al., 2014) may aid future review efforts.

### Strengths and Limitations

This review study was the first to systematically analyze published effectiveness studies of SDV prevention programs for male youth and linking the content of the programs and evaluated outcomes of the studies to the specific theoretical framework of the TPB. This resulted in a synthesized overview of what we do and do not know about the approaches and actual effectiveness of these programs. However, next to the limitations inherently arising from evaluation studies on programs related to SDV, such as self-report bias and selective drop-out, several limitations must be mentioned. First, we only selected studies that were published in English peer-reviewed journals. Although this provides the promise of including high-quality research, there is also a known barrier for public health-related research and research from low-income countries to get published in such journals (Adams et al., 2016). While this is a recurring issue to deal with in systematic review and meta-analyses, this may be particularly problematic in those that examine health program effectiveness studies. For example, in that field, RCT designs may be considered ‘the gold standard’, but its wide recognition has also been critiqued (Hein & Weeland, 2019). Specifically in dynamic and challenging real-world settings, there may be a lack of resources to properly conduct relatively costly and time-intensive studies like RCTs, in turn leading to difficulties publishing these studies in peer-reviewed journals. Hence, including also ‘grey literature” (e.g., dissertations, organization’s evaluation reports and pre-prints) could increase the quantity of the evidence for SDV prevention in male youth in terms of timelines (i.e., as peer-reviewed papers may take a long time to publish) and geographical locations in which studies were conducted (Batt et al., 2004). Secondly, we included only quantitative evaluation designs and based our conclusions on statistical effect sizes. However qualitative evaluations of SDV prevention programs–such as process evaluations or interviews–can provide insights in the experiences of participants and facilitators regarding the set-up, content and other characteristics of a program that promote, or hinder its implementation and effectiveness (for instance, see Freudberg et al., 2018). Thus, we strongly suggest that including qualitative program evaluation research may further advance our knowledge on the effective prevention of SDV, especially in terms of what works, for whom, and why.

### Conclusion

In this review of published quantitative effectiveness studies of group-focused, interaction based and multi-session SDV prevention programs for male youth, we observed that the combined body of evidence for such programs to change theoretically founded SDV-related predictors, is relatively small. Effects we found were mostly on behaviors (longer term) and attitudes (short-term), and whether these programs are also effective on the other relevant theoretical proxies of SDV, such as social norms and (perceived) behavioral control, remains largely unclear. Critics may argue that the overall relatively small effects beg the question to what extent these programs can be considered meaningfully effective, and when implementing such programs permits the substantive investment in terms of time and money. However, we would like to oppose that most importantly, there is still a lot of work to be done before such a conclusion can be validly drawn. Based on our evaluation of this body of literature, we have presented concrete suggestions for research and practice, and urge these fields to continue to collaborate toward continued program evaluation and further program development, using detailed descriptions of the programs and the evaluation designs. The formulation of relevant guidelines and effective program ingredients is an inherently iterative process, informed by theoretical perspectives and empirical insights, which are dynamic and subject to continuous change in youths’ social worlds. To conclude, investing in research and knowledge on effective early prevention of SDV across countries, and moreover, reducing the prevalence of SDV among youth worldwide, is of vital importance for both individual wellbeing and public health.

## Data Availability

The coding scheme is available as a supplementary material, and coded data are available upon request.

## Appendix 1

See Tables 6 and 7.

**Table 6:** Prisma abstracts checklist

Section and topic	Item No.	Checklist item	Reported (Yes/No)
Title			
Title	1	Identify the report as a systematic review	Yes
Background			
Objectives	2	Provide an explicit statement of the main objective(s) or question(s) the review addresses	Yes
Methods			
Eligibility criteria	3	Specify the inclusion and exclusion criteria for the review	Yes
Information sources	4	Specify the information sources (e.g. databases, registers) used to identify studies and the date when each was last searched	Yes
Risk of bias	5	Specify the methods used to assess risk of bias in the included studies	Yes
Synthesis of results	6	Specify the methods used to present and synthesis results	Yes
Results			
Included studies	7	Give the total number of included studies and participants and summarise relevant characteristics of studies	Yes
Synthesis of results	8	Present results for main outcomes, preferably indicating the number of included studies and participants for each. If meta-analysis was done, report the summary estimate and confidence/credible interval. If comparing groups, indicate the direction of the effect (i.e. which group is favoured)	Yes
Discussion			
Limitations of evidence	9	Provide a brief summary of the limitations of the evidence included in the review (e.g. study risk of bias, inconsistency and imprecision)	Yes
Interpretation	10	Provide a general interpretation of the results and important implications	Yes
Other			
Funding	11	Specify the primary source of funding for the review	N.A
Registration	12	Provide the register name and registration number	Yes

**Table 7:** Prisma Checklist

Section and topic	Item No.	Checklist item	Location where item is reported
Title			
Title	1	Identify the report as a systematic review	0 (title page)
Abstract			
Abstract	2	See the PRISMA 2020 for Abstracts checklist	p. 1
Introduction			
Rationale	3	Describe the rationale for the review in the context of existing knowledge	p. 8
Objectives	4	Provide an explicit statement of the objective(s) or question(s) the review addresses	p. 8
Methods			
Eligibility criteria	5	Specify the inclusion and exclusion criteria for the review and how studies were grouped for the syntheses	pp. 9–10
Information sources	6	Specify all databases, registers, websites, organisations, reference lists and other sources searched or consulted to identify studies. Specify the date when each source was last searched or consulted	p. 10
Search strategy	7	Present the full search strategies for all databases, registers and websites, including any filters and limits used	p. 59
Selection process	8	Specify the methods used to decide whether a study met the inclusion criteria of the review, including how many reviewers screened each record and each report retrieved, whether they worked independently, and if applicable, details of automation tools used in the process	p. 11
Data collection process	9	Specify the methods used to collect data from reports, including how many reviewers collected data from each report, whether they worked independently, any processes for obtaining or confirming data from study investigators, and if applicable, details of automation tools used in the process	p. 11
Data items	10a	List and define all outcomes for which data were sought. Specify whether all results that were compatible with each outcome domain in each study were sought (e.g. for all measures, time points, analyses), and if not, the methods used to decide which results to collect	p. 11
	10b	List and define all other variables for which data were sought (e.g. participant and intervention characteristics, funding sources). Describe any assumptions made about any missing or unclear information	pp. 11–13
Study risk of bias assessment	11	Specify the methods used to assess risk of bias in the included studies, including details of the tool(s) used, how many reviewers assessed each study and whether they worked independently, and if applicable, details of automation tools used in the process	p. 13
Effect measures	12	Specify for each outcome the effect measure(s) (e.g. risk ratio, mean difference) used in the synthesis or presentation of results	pp. 12–13
Synthesis methods	13a	Describe the processes used to decide which studies were eligible for each synthesis (e.g. tabulating the study intervention characteristics and comparing against the planned groups for each synthesis (item no. 5))	pp. 13–14
	13b	Describe any methods required to prepare the data for presentation or synthesis, such as handling of missing summary statistics, or data conversions	p. 13
	13c	Describe any methods used to tabulate or visually display results of individual studies and syntheses	p. 11
	13d	Describe any methods used to synthesize results and provide a rationale for the choice(s). If meta-analysis was performed, describe the model(s), method(s) to identify the presence and extent of statistical heterogeneity, and software package(s) used	p. 14
	13e	Describe any methods used to explore possible causes of heterogeneity among study results (e.g. subgroup analysis, meta-regression)	p. 13
	13f	Describe any sensitivity analyses conducted to assess robustness of the synthesized results	N.A
Reporting bias assessment	14	Describe any methods used to assess risk of bias due to missing results in a synthesis (arising from reporting biases)	p. 13
Certainty assessment	15	Describe any methods used to assess certainty (or confidence) in the body of evidence for an outcome	N.A
Results			
Study selection	16a	Describe the results of the search and selection process, from the **number of records identified** in the search **to the number of studies included in the review, ideally using a flow diagram**	p. 44
	16b	Cite studies that might appear to meet the inclusion criteria, but which were excluded, and explain why they were excluded	N.A
Study characteristics	17	Cite each included study and present its characteristics	pp. 14–15; 45–46
Risk of bias in studies	18	Present assessments of risk of bias for each included study	p. 54
Results of individual studies	19	For all outcomes, present, for each study: (a) summary statistics for each group (where appropriate) and (b) an effect estimate and its precision (e.g. confidence/credible interval), ideally using structured tables or plots	pp. 50–53
Results of syntheses	20a	For each synthesis, briefly summarise the characteristics and risk of bias among contributing studies	N.A
	20b	Present results of all statistical syntheses conducted. If meta-analysis was done, present for each the summary estimate and its precision (e.g. confidence/credible interval) and measures of statistical heterogeneity. If comparing groups, describe the direction of the effect	N.A
	20c	Present results of all investigations of possible causes of heterogeneity among study results	N.A
	20d	Present results of all sensitivity analyses conducted to assess the robustness of the synthesized results	N.A
Reporting biases	21	Present assessments of risk of bias due to missing results (arising from reporting biases) for each synthesis assessed	N.A
Certainty of evidence	22	Present assessments of certainty (or confidence) in the body of evidence for each outcome assessed	p. 18 – 22
Discussion			
Discussion	23a	Provide a general interpretation of the results in the context of other evidence	pp. 25–27
	23b	Discuss any limitations of the evidence included in the review	pp. 27–29
	23c	Discuss any limitations of the review processes used	pp. 29–30
	23d	Discuss implications of the results for practice, policy, and future research	pp. 27–31
Other information			
Registration and protocol	24a	Provide registration information for the review, including register name and registration number, or state that the review was not registered	p. 10
	24b	Indicate where the review protocol can be accessed, or state that a protocol was not prepared	p. 10
	24c	Describe and explain any amendments to information provided at registration or in the protocol	N.A
Support	25	Describe sources of financial or non-financial support for the review, and the role of the funders or sponsors in the review	p. 32
Competing interests	26	Declare any competing interests of review authors	p. 32
Availability of data, code and other materials	27	Report which of the following are publicly available and where they can be found: template data collection forms; data extracted from included studies; data used for all analyses; analytic code; any other materials used in the review	p. 32

## Appendix 2

See Table 8.

**Table 8:** Search terms used and filters applied for each database

Database	Dates	No. of results	Used syntax	Applied filters
Web of Science	12–3–21	7617	TS = ( ("sex* violen*" OR "sexual partner violence" OR SPV OR "sex* assault*" OR "sex* coerc*" OR rape OR rapist OR "Sexual* abus*" OR "Sex* harrass*" OR "sex* aggress*" OR "gender norm*" OR gender-norm* OR "gender-based violence" OR "gender* violence" OR "healthy masculinity" OR "dating violence" OR DV OR "teen dating violence" OR TDV OR "relation* abus*" OR "intimate partner violence" OR IPV OR "relation* violence" OR "relation* aggression" OR "physical* violen*" OR "physical* abus*" OR "emotional* abus*" OR "emotional* violen*" OR "couple violence" OR "couple aggression" OR "romantic violence" OR "romantic aggression" OR "dating aggression" OR "dat* abuse" OR “sex* victim”) AND (promotion OR intervention OR program* OR "Risk management" OR prevent*) AND (Effect* OR "Effect* of" OR Evaluat* OR Efficac* OR Outcome* OR chang* OR RCT OR "Controlled trial" OR Experiment OR "evidence based" OR "program* evaluation" OR test OR stud* OR assess* OR chang*) AND (Boys OR "Young people" OR High-school OR "teen*" OR Students OR "Young men" OR "Young male*" OR School-aged OR Adolescen* OR "grade" OR "Middle school" OR "Middle level" OR Youth OR "human males"))	Excluded: review (*n* = 603)
	9–3–22	891		
PsychInfo	23–3–21	7479	(("sex* violen*" or "sexual partner violence" or SPV or "sex* assault*" or "sex* coerc*" or rape or rapist or "Sexual* abus*" or "Sex* harrass*" or "sex* aggress*" or "gender norm*" or gender-norm* or "gender-based violence" or "gender* violence" or "healthy masculinity" or "dating violence" or DV or "dating abuse teen dating violence" or TDV or "relation* abus*" or "intimate partner violence" or IPV or "relation* violence" or "relation* aggression" or "physical* violen*" or "physical* abus*" or "emotional* abus*" or "emotional* violen*" or "couple violence" or "couple aggression" or "romantic violence" or "romantic aggression" or "dating aggression" or "sex* victimi*" or "dating abuse") and (promotion or intervention or program* or "Risk management" or prevent*) and (Effect* or Evaluat* or Efficac* or Outcome or "RCT" or "Controlled trial" or Experiment or "evidence based" or "program* evaluation" or test or study* or assess* or chang*) and (Boys or "Young people" or High-school or "teen*" or Students or "Young men" or "Young male*" or School-aged or Adolescen* or "grade" or "Middle school" or "Middle level" or Youth or "human males")).mp	N.A
	9–3–22	501		
PubMed	26–3–21	Search 1: 180 Search 2: 48 Total: 228	*Search 1:* (("sex* violen*" [Title/Abstract] OR "sexual partner violence" [Title/Abstract] OR SPV [Title/Abstract] OR "sex* assault*" [Title/Abstract] OR "sex* coerc*" [Title/Abstract] OR rape [Title/Abstract] OR rapist [Title/Abstract] OR "Sexual* abus*" [Title/Abstract] OR "Sex* harrass*" [Title/Abstract] OR "sex* aggress*" [Title/Abstract] OR "gender norm*" OR gender-norm* [Title/Abstract] OR "gender-based violence" [Title/Abstract] OR "gender* violence" [Title/Abstract] OR "healthy masculinity" [Title/Abstract] OR "dating violence" [Title/Abstract] OR DV [Title/Abstract] OR "dating abuse" [Title/Abstract] OR "teen dating violence" [Title/Abstract] OR TDV [Title/Abstract] OR "relation* abus*" [Title/Abstract] OR "intimate partner violence" [Title/Abstract] OR IPV [Title/Abstract] OR "relation* violence" [Title/Abstract] OR "relation* aggression" [Title/Abstract] OR "physical* violen*" [Title/Abstract] OR "physical* abus*" [Title/Abstract] OR "emotional* abus*" [Title/Abstract] OR "emotional* violen*" [Title/Abstract] OR "couple violence" [Title/Abstract] OR "couple aggression" [Title/Abstract] OR "romantic violence" [Title/Abstract] OR "romantic aggression" [Title/Abstract] OR "dating aggression" [Title/Abstract] OR "sex* victimi*" [Title/Abstract]) AND ("risk reduction" [Title/Abstract] OR promotion [Title/Abstract] OR intervention [Title/Abstract] OR program* [Title/Abstract] OR "Risk management" [Title/Abstract] OR prevent* [Title/Abstract]) AND (Effect* [Title/Abstract] OR Evaluat* [Title/Abstract] OR Efficac* [Title/Abstract] OR Outcome [Title/Abstract] OR RCT [Title/Abstract] OR "Controlled trial" [Title/Abstract] OR Experiment [Title/Abstract] OR "evidence based" [Title/Abstract] OR "program* evaluation" [Title/Abstract] OR test [Title/Abstract] OR study* [Title/Abstract] OR assess* [Title/Abstract] OR chang* [Title/Abstract]) AND (Boys [Title/Abstract] OR "Young people" [Title/Abstract] OR High-school [Title/Abstract] OR teen* [Title/Abstract] OR Students [Title/Abstract] OR "Young men" [Title/Abstract] OR "Young male*" [Title/Abstract] OR School-aged [Title/Abstract] OR Adolescen* [Title/Abstract] OR "grade" [Title/Abstract] OR "Middle school" [Title/Abstract] OR "Middle level" [Title/Abstract] OR Youth [Title/Abstract] OR "human males" [Title/Abstract])) *Search 2:* (("sex* violen*" [MeSH terms] OR "sexual partner violence" [MeSH terms] OR SPV [MeSH terms] OR "sex* assault*" [MeSH terms] OR "sex* coerc*" [MeSH terms] OR rape [MeSH terms] OR rapist [MeSH terms] OR "Sexual* abus*" [MeSH terms] OR "Sex* harrass*" [MeSH terms] OR "sex* aggress*" [MeSH terms] OR "gender norm*" OR gender-norm* [MeSH terms] OR "gender-based violence" [MeSH terms] OR "gender* violence" [MeSH terms] OR "healthy masculinity" [MeSH terms] OR "dating violence" [MeSH terms] OR DV [MeSH terms] OR "dating abuse" [MeSH terms] OR "teen dating violence" [MeSH terms] OR TDV [MeSH terms] OR "relation* abus*" [MeSH terms] OR "intimate partner violence" [MeSH terms] OR IPV [MeSH terms] OR "relation* violence" [MeSH terms] OR "relation* aggression" [MeSH terms] OR "physical* violen*" [MeSH terms] OR "physical* abus*" [MeSH terms] OR "emotional* abus*" [MeSH terms] OR "emotional* violen*" [MeSH terms] OR "couple violence" [MeSH terms] OR "couple aggression" [MeSH terms] OR "romantic violence" [MeSH terms] OR "romantic aggression" [MeSH terms] OR "dating aggression" [MeSH terms] OR "sex* victimi*" [MeSH terms]) AND ("risk reduction" [MeSH terms] OR promotion [MeSH terms] OR intervention [MeSH terms] OR program* [MeSH terms] OR "Risk management" [MeSH terms] OR prevent* [MeSH terms]) AND (Effect* [MeSH terms] OR Evaluat* [MeSH terms] OR Efficac* [MeSH terms] OR Outcome [MeSH terms] OR RCT [MeSH terms] OR "Controlled trial" [MeSH terms] OR Experiment [MeSH terms] OR "evidence based" [MeSH terms] OR "program* evaluation" [MeSH terms] OR test [MeSH terms] OR study* [MeSH terms] OR assess* [MeSH terms] OR chang* [MeSH terms]) AND (Boys [MeSH terms] OR "Young people" [MeSH terms] OR High-school [MeSH terms] OR teen* [MeSH terms] OR Students [MeSH terms] OR "Young men" [MeSH terms] OR "Young male*" [MeSH terms] OR School-aged [MeSH terms] OR Adolescen* [MeSH terms] OR "grade" [MeSH terms] OR "Middle school" [MeSH terms] OR "Middle level" [MeSH terms] OR Youth [MeSH terms] OR "human males" [MeSH terms]))	Excluded: Meta-analysis, Review, Systematic Review and Books & Documents
	9–3–22	Search 1: 27 Search 2: 1 Total: 28		
Scopus	19–4–21	7343	(TITLE-ABS ( "sex* violen*" OR "sexual partner violence" OR spv OR "sex* assault*" OR "sex* coerc*" OR rape OR rapist OR "Sexual* abus*" OR "Sex* harrass*" OR "sex* aggress*" OR "gender norm*" OR gender-norm* OR "gender-based violence" OR "gender* violence" OR "healthy masculinity" OR "dating violence" OR dv OR "dating abuse" OR "teen dating violence" OR tdv OR "relation* abus*" OR "intimate partner violence" OR ipv OR "relation* violence" OR "relation* aggression" OR "physical* violen*" OR "physical* abus*" OR "emotional* abus*" OR "emotional* violen*" OR "couple violence" OR "couple aggression" OR "romantic violence" OR "romantic aggression" OR "dating aggression" OR "sex* victimi*") OR AUTHKEY ( "sex* violen*" OR "sexual partner violence" OR spv OR "sex* assault*" OR "sex* coerc*" OR rape OR rapist OR "Sexual* abus*" OR "Sex* harrass*" OR "sex* aggress*" OR "gender norm*" OR gender-norm* OR "gender-based violence" OR "gender* violence" OR "healthy masculinity" OR "dating violence" OR dv OR "dating abuse" OR "teen dating violence" OR tdv OR "relation* abus*" OR "intimate partner violence" OR ipv OR "relation* violence" OR "relation* aggression" OR "physical* violen*" OR "physical* abus*" OR "emotional* abus*" OR "emotional* violen*" OR "couple violence" OR "couple aggression" OR "romantic violence" OR "romantic aggression" OR "dating aggression" OR "sex* victimi*")) AND ( TITLE-ABS ( promot* OR interven* OR program* OR "Risk reduction" OR prevent*) OR AUTHKEY ( promot* OR interven* OR program* OR "Risk reduction" OR prevent*)) AND ( TITLE-ABS ( effect* OR evaluat* OR efficac* OR outcome* OR rct OR "Controlled trial" OR experiment OR "evidence based" OR "program* evaluation" OR test OR study* OR assess* OR chang*) OR AUTHKEY ( effect* OR evaluat* OR efficac* OR outcome* OR rct OR "Controlled rial" OR experiment OR "evidence based" OR "program* evaluation" OR test OR study* OR assess* OR chang*)) AND ( TITLE-ABS ( boys OR "Young people" OR high-school OR teen* OR students OR "Young men" OR "Young male*" OR school-aged OR adolescen* OR "grade" OR "Middle school" OR "Middle level" OR youth OR "human males") OR AUTHKEY ( boys OR "Young people" OR high-school OR teen* OR students OR "Young men" OR "Young male*" OR school-aged OR adolescen* OR "grade" OR "Middle school" OR "Middle level" OR youth OR "human males"))	N.A
	9–3–22	1338		
Social Services Abstracts	25–3–21	1576	AB,TI,SU(sex* p/0 (violen* or assault* or coerc* or harrass* or aggress*) OR "sexual partner violence" OR SPV OR rape OR rapist OR Undesired p/0 sex* OR "unwanted sexual" p/0 experienc* OR Sexual*p/0 abus*) AND (promot* OR interven* OR program* OR "Risk management" OR prevent*) AND (Effect* OR Evaluat* OR Efficac* OR Outcome* OR RCT OR "Controlled trial" OR Experiment OR "evidence based" OR "program* evaluation" OR test OR study* OR assess* OR chang*) AND (Boys OR "Young people" OR High-school OR teen* OR Students OR "Young men" OR ("young male" OR "young males") OR School-aged OR Adolescen* OR "grade" OR "Middle school" OR "Middle level" OR Youth OR "human males")	Selected: ‘In scholarly journals’
	9–3–22	154		
ERIC	25–3–21	17	AB,TI,SU(sex* p/0 (violen* or assault* or coerc* or harrass* or aggress*) OR “sexual partner violence” OR SPV OR rape OR rapist OR Sexual* p/0 abus* OR gender p/0 norm* OR gender-norm* OR “gender-based violence” OR gender* p/0 violence OR "healthy masculinity" OR “dating violence” OR DV OR “dating abuse” OR “teen dating violence” OR TDV OR “relation* abus*” OR “intimate partner violence” OR IPV OR relation* p/0 (violence OR aggression) OR physical* p/0 (violen* OR abus*) OR emotional* p/0 (abus* OR violen*) OR couple p/0 (violence OR aggression) OR romantic p/0 (violence OR aggression) OR “dating aggression” OR “sex* victimi*”) AND AB,TI,SU(Effect* OR Evaluat* OR Efficac* OR Outcome* OR RCT OR "Controlled trial" OR Experiment OR "evidence based" OR "program* evaluation" OR test OR study* OR assess* OR chang*) AND AB,TI,SU(Effect* OR Evaluat* OR Efficac* OR Outcome* OR RCT OR "Controlled trial" OR Experiment OR "evidence based" OR "program* evaluation" OR test OR study* OR assess* OR chang*) AND AB,TI,SU(boy* OR male)	Grade 6 – 12 High school equivalency programs High schools Intermediate grades Junior high schools Middle schools Secondary education
	9–3–22	4		

## Appendix 3

See Table 9.

**Table 9:** Type of sexual and dating violence addressed in the program as described in the study, the used definition and operationalization (if any)

Study	Program	Type of SDV	Definition	Operationalization
Pulerwitz et al. (2015)	Breaking Gender Barriers	Physical or emotional violence against a female (partner or in general)	Physical or emotional violence against a female (partner or in general)	Female partner or non-partner physical or emotional violence
Miller et al. (2012, 2013, 2020b)	Coaching boys into men	Dating violence	Physical, sexual, and psychological aggression in adolescent romantic relationships	Eleven different abusive behaviors against a female partner
Santhya et al. (2019), Santhya and Zavier (2022)	Do Kadam	Violence against girls/women	–	Verbal sexual abuse (e.g. making dirty comments, teasing, spreading sexual rumors, etc.) against a girl. Pushing/grabbing or shoving a girl/woman. Assaulting or abusing a girl sexually or molesting her
Van Lieshout et al. (2019)	Make a move	Sexual harassment	Unwanted sexual comments, advances, or behaviors that cause harm to the victim, and sexual violence toward women	–
Pulerwitz et al. (2014)	Male Norms Initiative	Gender based violence and intimate partner violence	Physical, psychological, and sexual violence against female partner	Seven physically and sexually abusive behaviors and four psychologically violent behaviors against a woman
Miller et al. (2020a)	Manhood 2.0	Dating violence	Physical and sexual violence and psychological aggression in adolescent dating relationships	Twenty-seven different behaviors of sexual and dating violence, including cyber sexual abuse and sexual harassment
		Sexual Violence	Sexual harassment, sexual assault, and rape	
Miller et al. (2014)	Parivartan	Gender based violence	Including sexual harassment, sexual assault, and intimate partner violence	Six sexually abusive behaviors toward a female (not a family member)
Foley et al. (2015)	Program H	Gender based violence, sexual violence, and intimate partner violence	–	–
Powell-Williams et al. (2020)	Program H and Program M	Gender based violence, sexual violence, and intimate partner violence	–	–
De Graaf et al. (2016)	Rock and Water	Sexual aggression	Varying from non-consensual sexual touching to forced intercourse	Sexual Experience Survey (SES) and non-contact sexual aggression including sexual aggression
Banyard et al. (2019)	Reducing sexism and violence program	Sexual and dating violence, also referred to as gender-based violence	–	–
Gibbs et al. (2019)	Stepping stones and creating futures	Intimate partner violence	–	Physical and sexual partner violence (WHO instrument for violence against women, 4 items)
Keller et al. (2017)	Your moment of truth	Gender based violence	Including rape and sexual assault	Verbal harassment, physical threats, and physical and sexual assault
Freudberg et al. (2018)	Yuva Samaanta Ki Oor	Intimate partner violence	–	Behaviors related to gender, violence, and sexuality
