# Correction: Sexual and Dating Violence Prevention Programs for Male Youth: A Systematic Review of Program Characteristics, Intended Psychosexual Outcomes, and Effectiveness

**DOI:** 10.1007/s10508-023-02664-w

**Published:** 2023-07-24

**Authors:** Mirthe Verbeek, Joyce Weeland, Maartje Luijk, Daphne van de Bongardt

**Affiliations:** Youth and Family, Department of Child Development and Education, Erasmus University Rotterdam, P.O. Box 1738, 3000 DR Rotterdam, The Netherlands

**Correction to: Archives of Sexual Behavior** 10.1007/s10508-023-02596-5

Entries in the **References** that were missing for some articles cited in the article have been added since the original publication of this article.

The original article has been corrected.

